# A psychometric evaluation of the Caregiver Contribution to Self-Care of Heart Failure Index in a Thai population

**DOI:** 10.1186/s12955-021-01814-9

**Published:** 2021-07-10

**Authors:** Nittaya Srisuk, Nutchanath Wichit, David R. Thompson, Chantal F. Ski

**Affiliations:** 1 Faculty of Nursing, Surat Thani Rajabhat University, Surat Thani, Thailand; 2 School of Nursing and Midwifery, Medical Biology Centre, Queen’s University Belfast, 97 Lisburn Road, Belfast, BT9 7BL UK; 3 Integrated Care Academy, University of Suffolk, Ipswich, UK

**Keywords:** Psychometric evaluation, Caregiver Contribution to Self-Care to Heart Failure Index, Thai, Translation, Reliability, Validity

## Abstract

**Background:**

Caregivers are major contributor to the self-care of patients with heart failure. The Caregiver Contribution to Self-Care of Heart Failure Index (CC-SCHFI) measures these contributions across three scales: self-care maintenance (symptom monitoring and treatment adherence); self-care management (dealing with symptoms); and confidence in contributing to the self-care (self-efficacy in managing self-care) of patients with heart failure. Informal caregivers play a vital role in supporting family members with heart failure in Thailand, yet no validated tool exists to measure their contribution. We examined the psychometric properties of the CC-SCHFI in a Thai population.

**Methods:**

The CC-SCHFI was translated into Thai using a standard forward and backward translation procedure. A cross-sectional design was used to examine the psychometric properties of the Thai version of the CC-SCHFI in 100 family caregivers of heart failure patients in Southern Thailand. Confirmatory factor analysis was used to assess construct validity, and factor score determinacy coefficients were computed to evaluate internal consistency reliability.

**Results:**

The Thai version of the CC-SCHFI demonstrated acceptable internal consistency (composite reliability of each scale ranged from 0.76 to 0.99). Reliability estimates were adequate for each scale (McDonald’s omega ranged from 0.75 to 0.96). Confirmatory factor analysis supported the original factor structure of the instrument, with good fit indices for all three scales (comparative fit index = 0.98–1.00; root-mean-square error of approximation = 0.00–0.07).

**Conclusions:**

The Thai version of the CC-SCHFI appears to be a valid and reliable instrument for measuring caregiver contributions to self-care maintenance and self-care management as well as contributing to caregiver confidence in the self-care of Thai heart failure patients.

## Background

Heart failure is a complex life-limiting condition associated with a high rate of mortality, high symptom burden and poor quality of life. Globally, over 26 million people have heart failure [1]. The condition is worse in Thailand, where patients hospitalised for heart failure are younger and sicker, than Europe and the US [2]. Thailand, with a growing population of 70 million people, is categorized as an upper middle-income country [3]. As with other Southeast Asia countries, it has a high prevalence of cardiovascular disease risk factors, notably hypertension and raised blood glucose/diabetes, and thus of symptomatic heart failure [3]. Though data are sparse, in Thailand about 6% of people admitted with heart failure die in hospital [4]. One-year, five-year and ten-year mortality rates in Thai patients admitted for acute decompensated heart failure were 28%, 58% and 73%, respectively [5].

As the burden of heart failure increases in Thailand and other emerging economies, developing culturally appropriate, affordable and acceptable models is necessary [6]. This includes acknowledging the vital role family members play as informal caregivers of patients with heart failure in Thailand [7], a rapidly rising elderly population, and the burden imposed on caregivers [8, 9]. Such models are likely to be characterized by a systematic, coordinated and integrated approach to individual (patient/caregiver) assessment and intervention, ideally in the home or local community centre, but with access to specialist expertise at the local hospital or clinic. This will, of course, be determined by social, cultural and organizational structures and systems, such as beliefs and health insurance coverage, but aim to optimize health care and improve health outcomes and experience, confidence and satisfaction with care. Quality criteria should include equity and equality of opportunity to adherence to evidence-based assessments and treatments, counselling and health education, continuity and timely care, all underpinned by patient choice and preferences. Equally important is health care organized and delivered by competent, credentialed professionals with access to evidence-based guidelines and treatments [6, 10].

In Thailand, on initial admission and after diagnosis, patients with heart failure usually present with dyspnea, edema and weakness with common precipitating factors being noncompliance with diet and medication [11]; all preventable through appropriate self-care. Effective heart failure self-management strategies include adherence to complex medication regimens, exercise, daily weight and symptom monitoring and, where necessary, acting on these, as well as recommending other lifestyle behaviours [12]. Yet effective self-care practices, which can reduce re-hospitalization and mortality and enhance quality of life [13], is suboptimal among Thai patients with heart failure [14] with most requiring support from their family caregiver [15]. Further, although caregivers make an important contribution to the self-care of patients with heart failure, to date no instrument has been available to measure ‘caregiver contribution’ among patients with heart failure in Thailand.

The pivotal contribution of the caregiver to heart failure self-care in Western countries is well recognized, yielding improvements in health-related quality of life, re-hospitalization rates, adherence to treatment and engagement in self-care. Internationally, there is evidence that caregivers play a crucial role in supporting heart failure patients. For example, a recent systematic review and meta-analysis found that higher caregiver strain was associated with worse patient symptoms and quality of life [16]. The American Heart Association has indicated that it is critically important to understand the needs of caregivers to support the increasingly complex medical care they provide to patients living with heart failure [17]. In Thailand, where family members play a fundamental role in caring for their elders [8, 9], evidence pertaining to heart failure caregivers is limited, though it suggests that they are constrained in their caregiving role by factors such as inadequate knowledge and support, which can be improved by a simple family based-intervention [15]. The contribution of caregivers towards patients’ heart failure self-care is unclear due to the absence of a validated instrument for use in a Thai population.

In Western countries a number of instruments have been developed to measure caregiver contribution to heart failure self-care, including the Caregiver Contribution to Heart Failure Self-Care (CACHS) instrument [18], the Heart Failure Caregiver Questionnaire (HF-CQ) [19], the Caregiver Burden Questionnaire Heart Failure (CBQHF) [20] and the Caregiver Contribution to Self-Care to Heart Failure Index (CC-SCHFI) [21], recently revised [22], which is derived from the Self-Care of Heart Failure Index (SCHFI) [23] and has adequate reliability (Cronbach α > 0.80).

Of these instruments, only the SCHFI [24] is available in a Thai version. As the CC-SCHFI was derived from this, we aimed to translate and evaluate the psychometric properties of the CC-SCHFI in a sample of Thai speaking caregivers of patients with heart failure admitted to hospital in southern Thailand. Moreover, though the CC-SCHFI has been translated and used in Asian countries such as China, Korea, Japan, Vietnam and Indonesia which have similar, but not identical, social and cultural characteristics as Thailand, the Thai version of the CC-SCHFI may help provide nuanced insights into the role of heart failure caregivers in this population.

## Methods

### Study design

We conducted a cross-sectional study at two public hospital heart failure clinics in southern Thailand. A convenience sample of adult caregivers of patients with heart failure at each site was enrolled. All had been designated by patients as their primary caregivers and were older than 18 years of age, and able to read and write in Thai. All questionnaires were paper-based and self-administered. Data were collected between March to November 2020.

This study was approved by the Institutional Review Board of Suratthani Rajabhat University (SRU 2019_038). All research participants provided written informed consent and were informed of their right to withdraw anytime during the course of the study. The study conforms with the principles outlined in the Declaration of Helsinki.

### Instrument

The CC-SCHFI version 1 comprises 22 items divided across three scales: caregiver contribution to self-care maintenance; caregiver contribution to self-care management; and caregiver confidence in contributing to self-care [21]. The caregiver contribution to self-care maintenance scale comprises 10 items that measure caregiver contributions to symptom monitoring and treatment adherence. The caregiver contribution to self-care management scale comprises 6 items that measure caregiver ability to recognize symptoms of heart failure decompensation when they occur, to implement treatment in response to these symptoms, and to evaluate the treatments used. The caregiver confidence in contributing to self-care scale comprises 6 items that measure caregiver confidence in their skills in helping patients to engage in each phase of the self-care process. Each of the three scales uses a 4-point Likert response scale (for self-care maintenance ‘never or rarely, sometimes, frequently, always or daily’; for self-care management ‘not likely, somewhat likely, likely, very likely’; and for confidence in contributing to self-care ‘not sure, somewhat sure, sure, very sure’) with a standardized score from 0 to 100, higher scores indicating higher contribution to self-care [21].

### Instrument translation

The English version of the CC-SCHFI was forward and backward translated using the following techniques. Permission to use the CC-SCHFI was sought and obtained from the creator [21]. Two bilingual translators independently translated the instrument from English into the Thai language. This was followed by another review and verification by a bilingual (English and Thai) researcher and two translators who assessed the concepts and the appropriate use of language. At a later stage, two independent bilingual translators translated the Thai version of the CC-SCHFI back to English [25]. The translations were compared with the original to identify and amend any incorrect use of language and potential misinterpretations by the creators. The Thai version of the CC-SCHFI is provided in the “Appendix”.

### Sample size

Like a similar recent study evaluating the psychometric properties of the CC-SCHFI in a South American population [26], we determined a sample of seven participants per item was needed to allow adequate inference in confirmatory factor analysis [27]. As the CC-SCHFI has three separate scales, each measuring a different construct, with self-care maintenance being the longest scale, with 10 items, a sample of 70 participants would suffice to address dimensionality and internal consistency. However, as with the recent evaluation [26] that enrolled 99 participants to support a more stable analysis [28], we aimed to recruit a sample of 100 participants.

### Data analysis

Descriptive and inferential statistics were used to analyse the data. The Kaiser–Meyer–Olkin measure of sampling adequacy and Bartlett test of sphericity were used to assess data factorability. Confirmatory factor analysis (CFA) was used to test the construct validity of the Thai version of the CC-SCHFI and factor loading of the three scales. Maximum likelihood estimation was used to determine values for the parameters of a model [29]. Internal consistency reliability was tested by estimating item-to-total correlations using Spearman correlation coefficients. The item-to-scale correlation should exceed 0.4 [30]. Composite reliability (CR) of each latent variable and the average variance extracted (AVE) were calculated. McDonald’s omega coefficient was used to estimate the reliability [31, 32].

Similar to a previous study [26], we evaluated the following fit indices and criteria [33–39]: the comparative fit index (CFI), goodness of fit index (GFI), adjusted goodness of fit index (AGFI), incremental fit index (IFI); normed fit index (NFI), root mean square error of approximation (RMSEA), root mean square residual (RMSR). Regarding the CFI, values in the range of 0.90 to 0.95 indicate acceptable fit and values of 0.95 or higher indicate good fit [37]. The RMSEA was used to evaluate lack of model fit, where values of 0.05 or lower indicate a well-fitting model, values between 0.05 and 0.08 indicate moderate fit, and values of 0.10 or higher indicate poor fit. The RMSR was used to evaluate the fit in the sample, where values of 0.08 or lower indicate good fit.

## Results

### Sample characteristics

The sample comprised 100 caregivers of patients with heart failure (Table 1). Most caregivers were women (69%), with a mean age of 39 years; 51% were daughter or son.

**Table 1:** Sociodemographic characteristics of informal caregivers

Sociodemographic characteristics (n = 100)	n (%)
*Gender*	
Female	69
Age (mean, SD)	39.2 (8.5)
*Educational level*	
No formal education	22
Primary school	16
Secondary school/college	28
Bachelor or higher	34
*Occupation*	
Employee	24
Business owner/ trader	44
Farmer	15
Public employee	11
Unemployed/student	6
*Relationship with patient*	
Spouse	29
Daughter/son	51
Sister/brother	8
Other	12

### Item descriptive analysis

Table 2 shows descriptive statistics for the individual items of the CC-SCHFI. The highest-scoring items in the caregiver contribution maintenance scale were ‘keep doctor or nurse appointments’ and ‘eat a low salt’. Items related to ‘exercise for 30 min’ and ‘use a system (pill box, reminders) to help him/her remember medicines’ scored lowest. On the caregiver contribution to self-care management scale, the item that scored highest was ‘reduce the salt in diet’ whereas ‘take an extra water pill’ scored lowest. On the caregiver confidence in contributing to self-care scale, the highest-scoring item was ‘follow the treatment advice’. The lowest-scoring item was ‘prevent heart failure symptoms’.

**Table 2:** Descriptive statistics for individual items of the Thai version of the CC-SCHFI

Items	Min	Max	Mean	SD	Skewness	Kurtosis
*Caregiver contribution to self-care maintenance* *How often do you recommend that the person you care for do the following things?*						
1. Weigh daily	1	4	2.47	0.72	0.52	− 0.12
2. Check ankles for swelling	1	4	2.70	0.82	0.05	− 0.68
3. Try to avoid getting sick (e.g. flu shot, avoid ill people)	1	4	2.79	0.81	− 0.18	− 0.47
4. Do some physical activity	1	4	2.48	0.73	.038	− 0.19
5. Keep doctor or nurse appointments	3	4	3.80	0.40	− 1.52	0.32
6. Eat a low-salt diet	1	4	3.22	0.75	− 0.53	− 0.51
7. Exercise for 30 min	1	4	2.13	0.92	0.53	− 0.43
8. Remember to take medicines	1	4	3.04	1.13	− 0.68	− 1.04
9. Ask for low-salt items when eating out or visiting others	1	4	2.58	0.78	0.24	− 0.48
10. Use a system (pill box, reminder) to help him/her remember to take medicines?	1	4	2.23	0.85	0.33	− 0.40
*Caregiver contribution to self-care management*						
11. If the person you care for had trouble breathing or ankle swelling, how quickly did you recognize it as a symptom of heart failure? If the person you care for has trouble breathing or ankle swelling, how likely are you to recommend (or do) one of the following remedies?	1	4	2.13	0.99	0.50	− 0.77
12. Reduce salt in the diet	1	4	3.12	0.81	− 0.58	− 0.30
13. Reduce fluid intake	1	4	2.85	0.77	− 0.14	− 0.49
14. Take an extra water pill	1	5	1.54	0.94	0.22	0.78
15. Call your doctor or nurse for guidance	1	4	2.13	1.07	0.49	− 1.02
16. Think of a remedy you tried the last time the patient you care for care for had trouble breathing or ankle swelling. How sure were you that the remedy helped or did not help him or her?	0	4	2.34	0.89	− 0.12	− 0.48
*Caregiver confidence in contributing to self-care* *In reference to the person you care for, how confident are you that you can:*						
17. Prevent heart failure symptoms?	1	4	2.24	0.59	0.20	0.14
18. Follow the treatment advice?	2	4	2.80	0.62	0.16	− 0.50
19. Evaluate the importance of heart failure symptoms?	1	4	2.60	0.59	− 0.25	− 0.26
20. Recognize health changes in the person you care for?	1	4	2.48	0.61	0.08	− 0.30
21. Do something that relieves heart failure symptoms?	1	4	2.54	0.61	0.12	− 0.34
22. Evaluate how well a remedy works?	1	4	2.54	0.64	− 0.15	− 0.16

The Bartlett test of sphericity was significant (p < 0.0001), and the Kaiser–Meyer–Olkin index of sampling adequacy was 0.670. Based on these results, the data were suitable for factor analysis.

### Confirmatory factor analysis

**Fig. 1:**
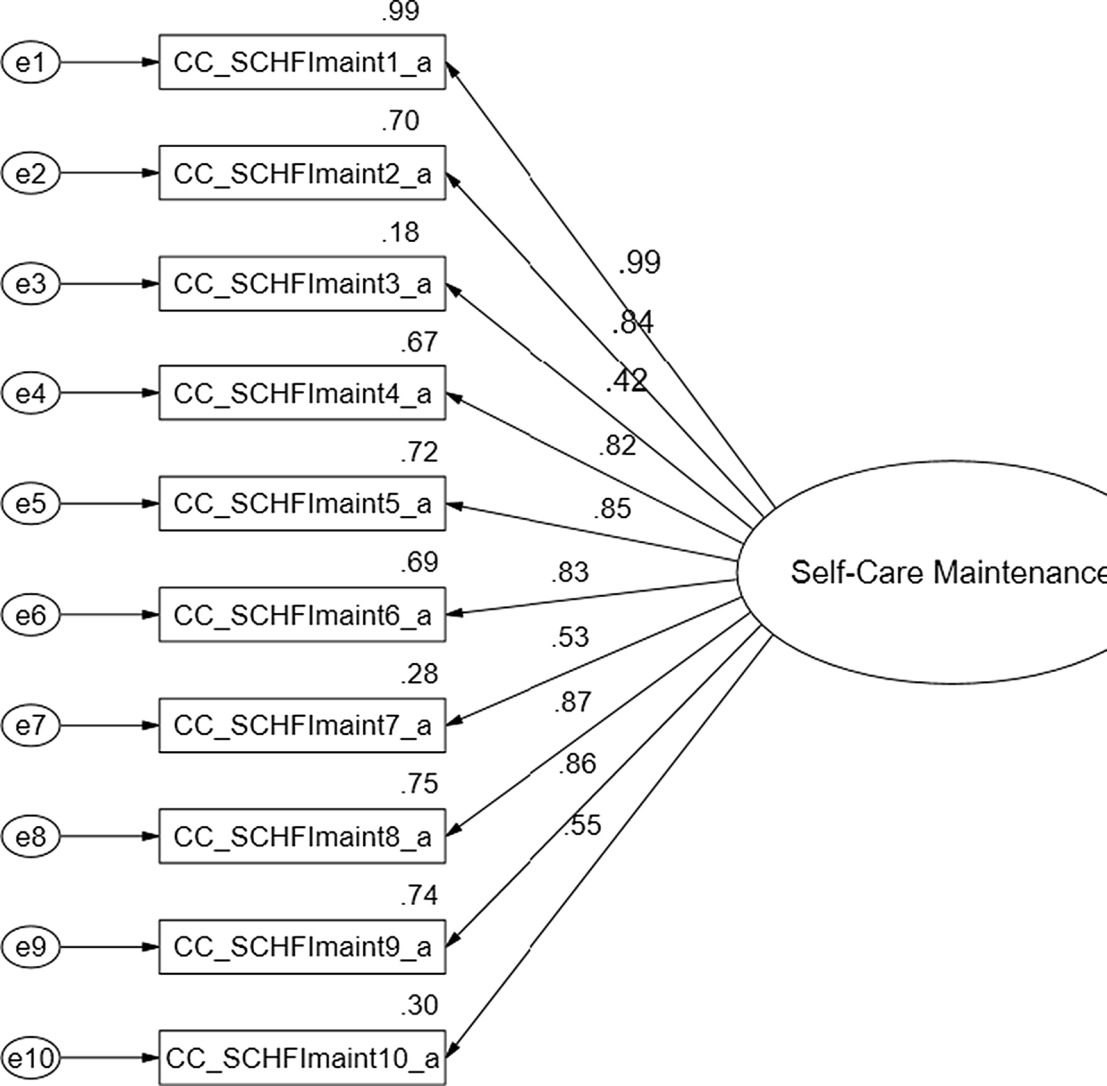
Confirmatory factor analysis of the Thai version of the CC-SCHFI caregiver contribution to self-care maintenance scale

**Fig. 2:**
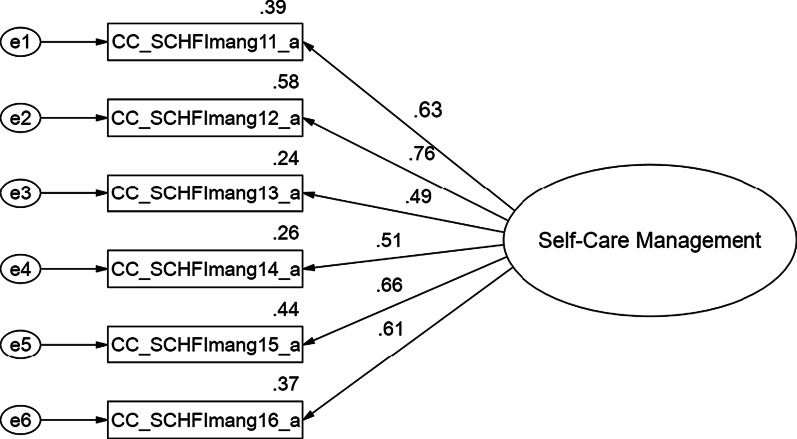
Confirmatory factor analysis of the Thai version of the CC-SCHFI caregiver contribution to self-care management scale

**Fig. 3:**
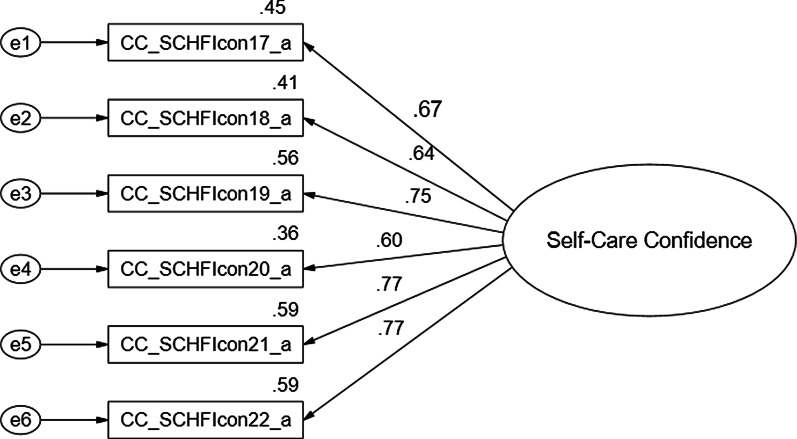
Confirmatory factor analysis of the Thai version of the CC-SCHFI caregiver confidence in contributing to self-care scale

**Fig. 4:**
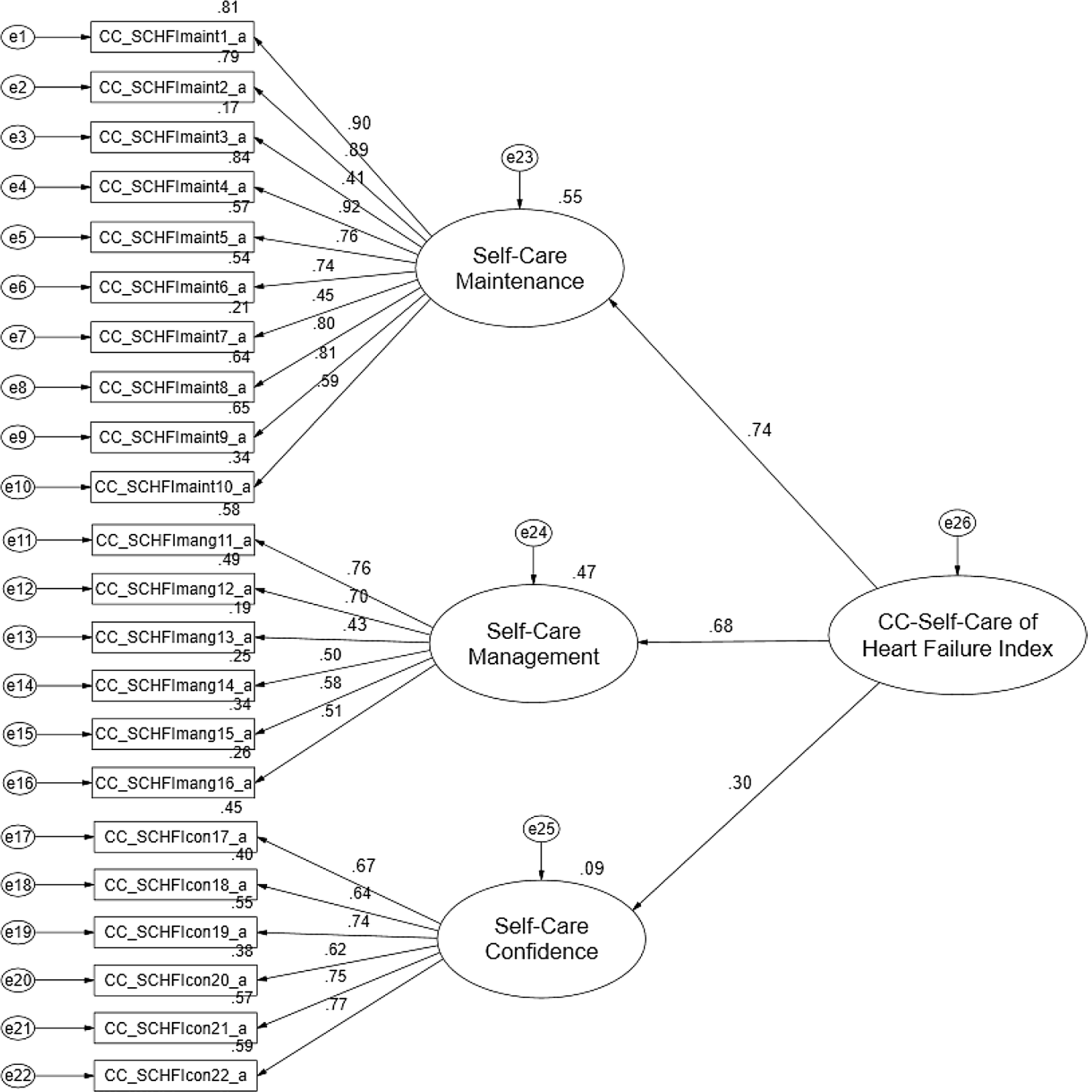
Confirmatory factor analysis of the Thai version of the CC-SCHFI

Findings of the CFA for each of the three CC-SCHFI scales and the index are presented below and in Figs. 1, 2, 3 and 4.

### Caregiver Contribution to Self-care Maintenance Scale

The self-care maintenance variables (10 latent variables) show acceptable threshold levels, Chi-square = 18.41, df = 15.0, *p* = 0.24, CMIN/df = 1.23 < 2.0, consistent with the concept [33–39], and a reasonable fit of the index of model to the data on the basis of a number of fit statistics, including CFI = 0.99, GFI = 0.97, AGFI = 0.87, RMSEA = 0.05, RMSR = 0.02, NFI = 0.90 and IFI = 0.99. Squared Multiple Correlations (R^2^) ranged from 18% – 99%, and the standardized factor loadings ranged from 0.42 – 0.99. The CFA of the self-care maintenance model strongly suggests that each set of items represents a single underlying construct and provides evidence for discriminate validity or acceptable fit (Fig. 1).

### Caregiver Contribution to Self-care Management Scale

The self-care management variables (6 latent variables) show acceptable threshold levels, Chi-square = 2.41, df = 3.0, *p* = 0.49, CMIN/df = 0.80 < 2.0, consistent with the concept [33–39], and a reasonable fit of the index of model to the data on the basis of a number of fit statistics including CFI = 1.00, GFI = 0.99, AGFI = 0.95, RMSEA = 0.00, RMSR = 0.02, NFI = 0.99, and IFI = 1.00. R^2^ ranged from 26% – 58%, and the standardized factor loading ranged from 0.49 – 0.76. The CFA of the self-care management model strongly suggests that each set of items represents a single underlying construct and provides evidence for discriminate validity or acceptable fit (Fig. 2).

### Caregiver Confidence in Contributing to Self-care Scale

The self-care confidence variables (6 latent variables) show acceptable threshold levels, Chi-square = 13.580, df = 9.0, *p* = 0.138, CMIN/df = 1.509 < 2.0, consistent with the concept [33–39], and a reasonable fit of the index of model to the data on the basis of a number of fit statistics including CFI = 0.98, GFI = 0.96, AGFI = 0.90, RMSEA = 0.05, RMR = 0.02, NFI 0.94, and IFI = 0.98. R^2^ ranged from 36% – 59%, and the standardized factor loading ranged from 0.60 – 0.77. The CFA of the self-care confidence model strongly suggests that each set of items represents a single underlying construct and provides evidence for discriminate validity or acceptable fit (Fig. 3).

### Caregiver Contribution to Self-Care of Heart Failure Index

The model for the Thai version of the CC-SCHFI (Fig. 4) identified the three latent variables of self-care maintenance, self-care management and self-care confidence. R^2^ across the three scales ranged from 17 to 84%, and the standardized factor loading ranged from 0.41 to 0.92 more than 0.40. The average variance extracted, which measures the variance captured by the indicators relative to measurement error, ranged from 0.52 to 0.59 and composite reliability ranged from 0.76 to 0.99 [40, 41]. McDonald’s omega ranged from 0.75 to 0.96 [31, 32] (Table 3). Item—total correlations ranged from 0.42 to 0.67. These acceptable values strongly suggest that each set of items represents a single underlying construct and provides evidence for discriminate validity. For each of the three scales an acceptable model fit was demonstrated based on a number of fit statistics (Table 4).

**Table 3:** Composite reliability, McDonald’s omega and average variance extracted for each CC-SCHFI scale

CC-SCHFI scale	CR	McDonald’s omega	AVE
Self-care maintenance	0.992	0.963	0.557
Self-care management	0.757	0.751	0.515
Self-care confidence	0.852	0.856	0.592

*CR* Composite reliability, *AVE* Average variance extracted

**Table 4:** Fit indices for each scale of the Thai version of the CC-SCHFI

CC-SCHFI scale	Chi-square/df	Sig	CMIN/df	GFI	AGFI	NFI	IFI	CFI	RMR	RMSEA
Maintenance	18.409/15.0	0.242	1.227	0.965	0.873	0.986	0.997	0.997	0.021	0.048
Management	2.416/3.0	0.491	0.805	0.992	0.944	0.992	1.002	1.000	0.016	0.000
Confidence	13.580/9.0	0.138	1.509	0.958	0.903	0.941	0.979	0.976	0.022	0.050

*GFI* Goodness of Fit Index, *AGFI* adjusted Goodness of Fit Index, *NFI* Normed Fit Index, *IFI* Incremental Fit Index, *CFI* Comparative Fit Index, *RMR* root mean square residual, *RMSEA* Root Mean Square Error of Approximation

For the model of the CC-SCHFI, the scale with the most influence was self-care maintenance (factor loading = 0.74; R^2^ = 55%), followed by self-care management (factor loading = 0.68; R^2^ = 47%) and lastly self-care confidence (factor loading = 0.30; R^2^ = 9%) (Fig. 4).

## Discussion

In this study, we tested the psychometric properties of the Thai version of the CC-SCHFI. To our knowledge, this is the first study to do so. Overall, our results show that the CC-SCHFI is a valid and reliable instrument to measure caregiver contributions to self-care of Thai heart failure patients.

The dimensionality of the CC-SCHFI was determined by performing a CFA of each of the three scales of the index: caregiver contribution to self-care maintenance (symptom monitoring and treatment adherence); caregiver contribution to self-care management (dealing with symptoms); and caregiver confidence in contributing to self-care (self-efficacy in managing self-care). Factor analysis was also performed on the entire CC-SCHFI and demonstrated an overall acceptable model fit to the data. The goodness-of-fit indices supported the hypothesized models from the original CC-SCHFI [21, 26].

As demonstrated in the original study [21], we found the Thai version of the CC-SCHFI had a complex structure that encompassed several distinct aspects related to self-care. The caregiver contribution to self-care management and the caregiver confidence in contributing to self-care scales demonstrated high factor loadings within each scale, attesting to a substantial proportion of common variance among the items. Only two items had low factor loadings on the caregiver contribution to self-management scale, ‘Try to avoid getting sick’, and ‘Use a system (pill box, reminder) to help him/her remember to take medicines’, which may be explained in part by social and cultural factors, such as the use of pill boxes in rural Thailand being uncommon. Of the three scales, the caregiver confidence in contributing to self-care one was the only scale to have a low factor loading for the overall index, which may again be explained by family, social and cultural structures and mores typical of rural Thailand, such as an essentially patriarchal family structure, with households generally deferring to the eldest members. This may also be compounded by issues such as the likelihood of low literacy levels, lack of health insurance and limited access to health care in Thailand [6, 8], particularly in rural and remote regions. Thus, for example, it may be incongruent for a child or a female caregiver to gain confidence in contributing to the self-care of a parent or male, often both.

Our findings are in general agreement with those of the original studies which were conducted with Italian [21] and Brazilian [26] samples. The finding that the self-care maintenance scale was the most influential is perhaps explained by caregivers, with limited access to heart failure services, playing a major role in symptom monitoring and treatment adherence by, for example, encouraging patients to take exercise, reminding them to attend clinic appointments and ensuring they weigh themselves and follow a recommended diet.

The CC-SCHFI serves as a practical tool for measuring caregiver contributions to heart failure self-care: it can aid caregivers in identifying gaps in heart failure self-care and in indicating measures to address them. The CC-SCHFI can also assist healthcare providers in guiding and supporting the skills development of caregivers. As self-care is a continuing process, the instrument can be used to monitor individual progress and benchmark this with that of others. It can also act as a motivating force to accomplish self-care.

A strength of our study is the translation and validation of an instrument, hitherto unavailable, to aid informal caregivers in the self-care of Thai people with heart failure. Specifically, the CC-SCHFI provides a comprehensive evaluation of caregiver contributions to self-care maintenance, management and confidence, and aids caregivers in identifying potential gaps in these three domains. Currently, no scale in Thai exists that measures ‘caregiver contribution’ to patients with heart failure or any long-term condition. ‘Caregiver burden’ has been measured, but this is not a comparable construct. As such, future studies should evaluate the concurrent validity of the Thai version of the CC-SCHFI with the Thai version of the SCHFI. The SCHFI might be used to determine criteria outcomes, i.e. whether caregiver intervention for the patient is reflected in the latter’s ability to care for themself and, hence, a suitable score on the SCHFI.

There are some limitations to our study that should be noted. First, only caregiver, not patient, self-care data were obtained, so we are unable to determine impact of caregiver contributions to heart failure patient self-care. Second, data were collected on one occasion only, so we are unable to estimate scale item stability. Third, in the absence of a well-established comparable measure, we were unable to determine concurrent validity. Fourth, our sample size may be deemed small, but as CFA recommendations for sample size-to-item ratio range vastly from 3–20 with absolute ranges with a minimum of 100 [42], we believe it to be acceptable. Moreover, it is comparable to the sample (n = 99) reported in the study validating the CC-SCHFI in a South American population [26]. For SEM, again this varies, with rules of thumb of 10 cases/observations per indicator variable in setting a lower bound of an adequate sample size, though the ratio can be as low as 5 to 1 [43]. Finally, shortly after commencing this study, a revised version of the CC-SCHFI [22] was published, and a translation and psychometric evaluation of this is warranted.

## Conclusions

The Thai version of the CC-SCHFI is a reliable and valid instrument to measure the contributions of caregivers to the self-care of patients with heart failure. This is the first instrument of its kind for use in a Thai population and appears to be useful for clinical and research purposes. Further studies to examine the use of this version of the instrument among other populations and settings are warranted. For example, consideration may be given to using the actor- interdependence model to estimate different dyadic patterns.

## Data Availability

The datasets used and/or analyzed during the present study and the Thai version of the CC-SCHFI are available from the corresponding author on reasonable request.

## Abbreviations

CC-SCHFI: Caregiver Contribution to Self-Care of Heart Failure Index (CC-SCHFI)
CFA: Confirmatory factor analysis
CFI: Comparative fit index
GFI: Goodness of fit index
AGFI: Adjusted goodness of fit index
IFI: Incremental fit index
NFI: Normed fit index
RMSEA: Root mean square error of approximation
RMSR: Root mean square residual
